# Cancer Alters the Metabolic Fingerprint of Extracellular Vesicles

**DOI:** 10.3390/cancers12113292

**Published:** 2020-11-06

**Authors:** Mari Palviainen, Kirsi Laukkanen, Zeynep Tavukcuoglu, Vidya Velagapudi, Olli Kärkkäinen, Kati Hanhineva, Seppo Auriola, Annamari Ranki, Pia Siljander

**Affiliations:** 1 EV group, Molecular and Integrative Biosciences Research Programme, Faculty of Biological and Environmental Sciences, University of Helsinki, 00790 Helsinki, Finland; mari.palviainen@helsinki.fi (M.P.); zeynep.tavukcuoglu@helsinki.fi (Z.T.); 2 CURED, Drug Research Program, Faculty of Pharmacy, Division of Pharmaceutical Biosciences, University of Helsinki, 00790 Helsinki, Finland; 3 EV-core, Faculty of Biological and Environmental Sciences, University of Helsinki, 00790 Helsinki, Finland; 4 Department of Dermatology and Allergology, Clinicum, University of Helsinki and Helsinki University Hospital, 00290 Helsinki, Finland; kirsi.laukkanen@helsinki.fi (K.L.); annamari.ranki@hus.fi (A.R.); 5 Metabolomics Unit, Institute for Molecular Medicine Finland (FIMM), University of Helsinki, 00290 Helsinki, Finland; viddu.vtt@gmail.com; 6 LC-MS Metabolomics Center, School of Pharmacy, University of Eastern Finland, 70211 Kuopio, Finland; olli.karkkainen@uef.fi (O.K.); seppo.auriola@uef.fi (S.A.); 7 Institute of Public Health and Clinical Nutrition, University of Eastern Finland, 70211 Kuopio, Finland; kati.hanhineva@utu.fi; 8 Food Chemistry and Food Development unit, University of Turku, 20500 Turku, Finland

**Keywords:** extracellular vesicles, cancer metabolism, cutaneous T-cell lymphoma, prostate cancer, colon cancer

## Abstract

**Simple Summary:**

Cancer changes cell metabolism. In this study, we explored if the metabolic rewiring also alters the metabolite content of cancer-derived extracellular vesicles (EVs). For this, metabolomes of EVs from different cancers (prostate, cutaneous T-cell lymphoma, and colon cancer cell lines) were compared with the metabolomes of control EVs derived from matched non-cancerous cell lines. The metabolomes of EVs from all three cancer types significantly differed from their respective control EVs by elevated levels of proline and succinate. Additionally, prostate and cutaneous T-cell lymphoma cell line –derived EVs contained elevated levels of creatinine and folate when compared to controls. In conclusion, this study presents the first evidence that a shared panel of metabolites in EVs reflects the altered metabolic state of multiple cancer cell types in vitro. These results warrant further studies of the significance and usability of a metabolic fingerprint in cancer studies and for biomarker discovery.

**Abstract:**

Cancer alters cell metabolism. How these changes are manifested in the metabolite cargo of cancer-derived extracellular vesicles (EVs) remains poorly understood. To explore these changes, EVs from prostate, cutaneous T-cell lymphoma (CTCL), colon cancer cell lines, and control EVs from their noncancerous counterparts were isolated by differential ultracentrifugation and analyzed by nanoparticle tracking analysis (NTA), electron microscopy (EM), Western blotting, and liquid chromatography-mass spectrometry (LC-MS). Although minor differences between the cancerous and non-cancerous cell-derived EVs were observed by NTA and Western blotting, the largest differences were detected in their metabolite cargo. Compared to EVs from noncancerous cells, cancer EVs contained elevated levels of soluble metabolites, e.g., amino acids and B vitamins. Two metabolites, proline and succinate, were elevated in the EV samples of all three cancer types. In addition, folate and creatinine were elevated in the EVs from prostate and CTCL cancer cell lines. In conclusion, we present the first evidence in vitro that the altered metabolism of different cancer cells is reflected in common metabolite changes in their EVs. These results warrant further studies on the significance and usability of this metabolic fingerprint in cancer.

## 1. Introduction

In cancer, activated protein products of oncogenes and mutant tumor suppressor genes can modify various metabolic pathways. The altered metabolic pathways allow tumor cells to better take up nutrients, generate biosynthetic precursors and macromolecules, and tolerate redox stress and hypoxia, thus supporting their proliferation and survival [1,2]. Such deregulation of cellular energetics is considered as one of the hallmarks of cancer [3]. The best-characterized metabolic phenomenon in cancer is the “Warburg effect”, namely aerobic glycolysis where glucose is converted to lactate even when oxygen is present. The excess of cellular lactate is secreted to the microenvironment, leading to acidification or incorporation into biomass which in turn facilitates rapid cell division [4].

Oncometabolites are common cell metabolites that aberrantly accumulate in cancers compared to non-proliferating cells and have pro-oncogenic functions. Consequently, metabolites produced from altered metabolic pathways and the dysregulated enzymes producing them present attractive biomarkers and therapeutic targets for cancer treatment [5]. Thus far, four oncometabolites (succinate, D-2-hydroxyglutarate, L-2-hydroxyglutarate, and fumarate) have been identified. Accumulation of these oncometabolites in cancer cells leads to metabolic and epigenetic alterations, post-translational modifications, and other pro-tumorigenic functions [6].

EVs are lipid bilayer encapsulated nanovesicles with multimolecular cargo that participate in intercellular communication. EVs harbor nucleic acids, proteins, lipids, and metabolites derived from their parent cell of origin [7,8], but can also carry on their surface extravesicular cargo [9]. In cancer, EVs have been shown to mediate intercellular transfer of these molecules between the different cells at the primary tumor site, but also systemically between organs [10]. Thus, EVs are considered to participate in cancer progression by modulating angiogenesis, immune cell function, thrombosis, epithelial to mesenchymal transition, and pre-metastatic niche formation [11,12]. A recent study by Hoshino et al. [13] identified novel cancer biomarkers from tumor tissue- and plasma-derived EVs using proteomics and machine learning, confirming that EVs are a potential source of cancer biomarkers.

The majority of the EV cargo studies have focused on proteins and nucleic acids; only a few studies have addressed metabolite content (reviewed in [14]). The potential of the EV metabolome as a source for biomarker discovery was first shown by comparative metabolomics of plasma-derived EVs from endometrial cancer patients and healthy controls, which distinguished these two groups from each other [15]. Another study showed that the alteration of metabolite profiles of urine-derived EVs could be associated with the severity of prostate cancer (PCa), suggesting that EV metabolomics could offer a new tool for the detection of PCa [16]. In another supporting study, a comparison of urinary EVs obtained from patients with PCa or benign prostate hyperplasia revealed 76 significantly altered metabolites, from which five exhibited differential abundance in high-grade tumors [17]. Furthermore, a comparison of EVs of aggressive and non-aggressive hepatocellular carcinoma cell lines showed that EVs from the aggressive cell lines were enriched in molecules for glycolysis I, gluconeogenesis, and pentose phosphate pathways [18]. These pioneering studies suggest that the altered metabolism of the tumor is mirrored in the cancer-derived EVs, and thus EV metabolites are not only a promising source of biomarkers but could also help elucidate the molecular basis of cancer progression.

In this study, we explored whether any of the metabolome changes in EVs would be shared irrespective of cancer type. Cell lines chosen for this study represented metastatic prostate carcinoma, primary cutaneous anaplastic large cell lymphoma (pcALCL), a subtype of cutaneous T-cell lymphoma (CTCL), and colon carcinoma. The majority of prostate cancers are carcinomas, which originate from epithelial cells. Despite the high survival rate in localized prostate cancer, metastatic prostate cancer remains largely incurable [19]. CTCLs are a heterogeneous group of non-Hodgkin lymphomas, characterized by a malignant population of mature T lymphocytes homing to the skin. The clinical course of CTCL varies greatly depending on the subtype [20]. Most colon carcinomas are adenocarcinomas that originate from epithelial cells of the colon mucosa [21]. We utilized two different metabolomic approaches. We used liquid chromatography-mass spectrometry (LC-MS) to identify changes in the metabolite cargo of cancer cell-line derived EVs to detect cancer-related metabolite signatures. First, an untargeted approach was applied to reveal differences between prostate cancer cell -derived EVs and noncancerous EVs. Next, we analyzed the metabolites of EVs from two different cancer types and their respective control EVs in a targeted manner. Despite the many differences in the cancer types, our data revealed that metabolomics can identify common cancer-associated metabolite cargo changes in EVs. Interestingly, succinate (a well-known oncometabolite [22]) and proline (a key metabolite whose pathway supports cancer cell survival, proliferation, and metastasis [23]) were upregulated in all studied cancer cell line -derived EVs compared to controls. Additionally, folate (a metabolite that supports enhanced DNA synthesis in cancers [24]) and creatinine (a byproduct of ATP production from phosphocreatine [25]) were shared upregulated metabolites in prostate carcinoma and CTCL cell line -derived EVs.

## 2. Results

### 2.1. Characterization of EVs

**Figure 1:**
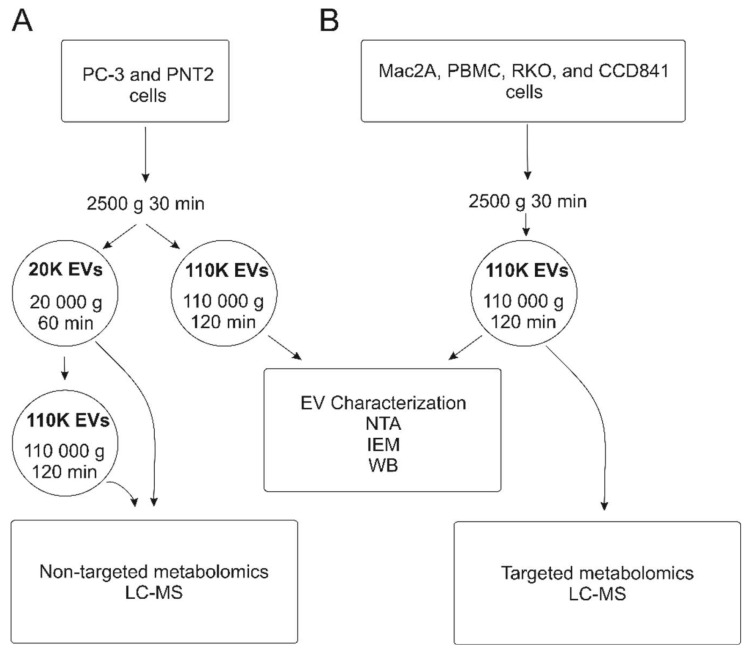
Study workflow. Two separate studies were performed: (**A**) extracellular vesicles (EVs) were isolated from prostate carcinoma cell line PC-3 and its control counterpart cell line PNT2 with 20,000× *g* (20 K, *n* = 3) and 110,000× *g* (110 K, *n* = 3) ultracentrifugation, and non-targeted LC-MS analysis of polar and non-polar analytes were performed. (**B**) Next, a targeted LC-MS analysis of polar analytes was performed for EVs isolated from the CTCL cell line Mac-2A, control peripheral blood mononuclear cells, colon carcinoma, and normal colon epithelial cell cultures (RKO and CCD841, respectively) with 110 K ultracentrifugation (*n* = 3). PC3 = prostate carcinoma; PNT2 = normal prostate epithelial cells; Mac-2A = cutaneous T-cell lymphoma; PBMC = peripheral blood mononuclear cells; RKO = colon carcinoma; CCD841 = normal colon epithelial cells; LC-MS = Liquid chromatography-mass spectrometry; WB = Western blot; NTA = Nanoparticle Tracking analysis; IEM = immunoelectron microscopy.

**Figure 2:**
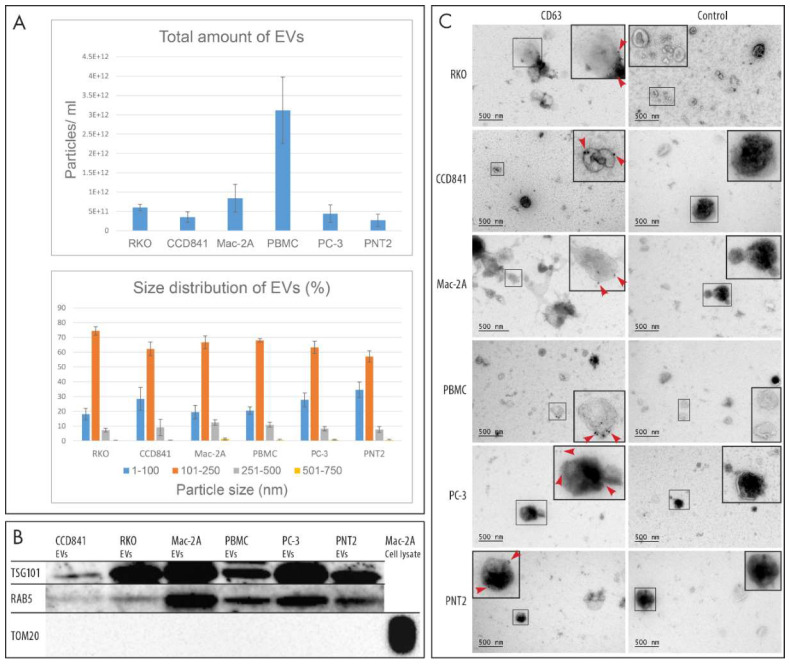
Characterization of EVs. (**A**) Nanoparticle tracking analysis of total amount and size distribution of EVs. Four replicates of each EV type were analyzed. (**B**) Western blot analysis of TSG101 and RAB5 (conventional EV markers) revealed that these markers were present in all EV samples, while the mitochondrial protein TOM20 was present only in the cell lysate used as a control. Total of 1.0E10 EVs were loaded in each well. (**C**) Immunoelectron microscopy revealed the presence of the EV marker CD63 on the surface of EVs by 10-nm colloidal gold particles (red arrowheads). Immunostaining without the primary antibody was used as a negative control for CD63 immunoelectron microscopy.

In this study, we sought to analyze whether any shared metabolite changes could be found from the EVs of prostate carcinoma, colon carcinoma, and T-cell lymphoma cell lines to identify the cancerous vs. non-cancerous state of cells when compared to their benign counterparts. To achieve this, EVs were isolated according to previously established methodologies and characterized for particle number, size, morphology, and typical EV protein signatures according to the MISEV guidelines [8]. A flowchart of the study is presented in Figure 1. According to Nanoparticle tracking analysis (NTA), prostate and colon carcinoma cell lines yielded more EVs than their normal cell line-derived counterparts. However, fewer EVs were generated from CTCL cells than from equal numbers of peripheral blood mononuclear cells (Figure 2A). No differences in EV size were observed between the six cellular sources of EVs, and most of the EVs were 101–250 nm in size (Figure 2A). Common EV markers, the cytosolic protein TSG101, and the transmembrane protein RAB5 were present in all EVs when analyzed by Western blot (equal loading of 1.0E10 EVs per well). The mitochondrial protein TOM20, a negative control, was only detected in cell lysates (Figure 2B, original WB figures in Supplementary Materials Figure S1). The presence of the transmembrane EV marker, CD63, was observed on the surface of EVs by immunoelectron microscopy (IEM). IEM also confirmed the typical EV morphology (Figure 2C).

### 2.2. Non-Targeted Metabolite Profile Analysis of EVs from Prostate Cancer PC-3 and Normal Prostate Epithelial PNT2 Cell Lines

**Figure 3:**
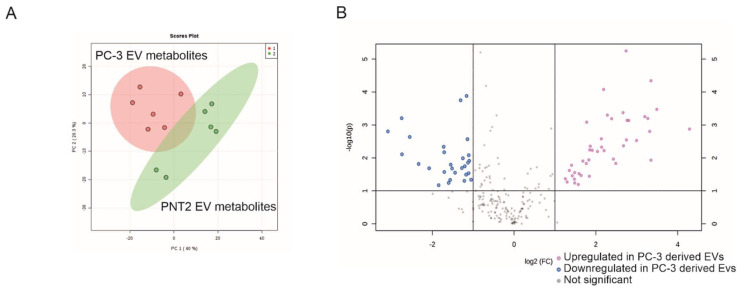
Metabolites differentially packed into EVs by the cancerous vs. non-cancerous prostate cell lines (*n* = 6). (**A**) Principal component analysis (PCA) of the LC-MS identified metabolites according to the cell lines of EV origin. For PCA, the metabolites of the 20K and 110K EV subpopulations from PC-3 and PNT2 cell lines were combined. (**B**) Volcano plot of the different metabolites in EVs (20K and 110K EVs combined) derived from PC-3 and PNT2 -cell lines.

We first investigated if EVs derived from cancer cells carried any metabolic cargo distinguishable from the metabolites in EVs derived from non-cancerous cells. To test this, we chose the PC-3 prostate cancer cell line as a model for cancerous cells and PNT2 for normal cells, as we had previous evidence for altered nucleic acid cargo in the EVs from these two cell lines [26,27]. The EVs derived from PC-3 and PNT2 cells were subjected to non-targeted metabolomics to obtain broad-spectrum metabolite coverage to reveal any differences. For the same reason, large (20K pellet from ultracentrifugation) and small (110K pellet) EVs were isolated separately by differential ultracentrifugation to reveal any population differences. From the LC-MS analysis of these EV samples, more than 6000 molecular features were extracted; 197 of these features were identified (Table S1). The results of the PC-3 derived EVs have been previously reported [28]. As the comparison of the 20K and 110K population did not show major differences in metabolites, the data were combined to analyze the entire EV metabolome and to compare the differences between PC-3 and PNT2. Principal component analysis (PCA) of the combined 20K and 110K samples revealed a clear separation between PC-3 and PNT2 cell line-derived EVs (Figure 3A). Among the 197 identified metabolites, 66 were metabolites of interest based on their significance versus fold-change values (Figure 3B). Forty-one metabolites were upregulated (FC < 2, *p* ≤ 0.05) in the EVs derived from the PC-3 prostate cancer cell line, and 25 in the normal prostate epithelial PNT2 cell line. Since ultracentrifugation is known to also co-isolate protein aggregates and lipoproteins as contaminants [29,30], we also compared each cell culture medium to determine if the differences could due to the media formulations. A comparison of the media did not reveal major differences between components identified in this study as metabolites of interest (Table S2).

**Figure 4:**
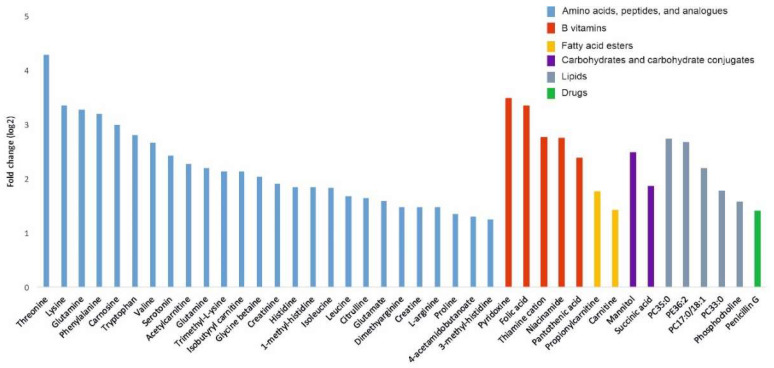
Bar graph of upregulated metabolites (log2 fold change ≥ 1.2) based on their significance versus fold-change values in PC-3-derived EVs compared to PNT2-derived EVs (*n* = 6).

Further analysis of the identified metabolite species revealed that the majority of the upregulated metabolites from the PC-3 cell-derived EVs were polar (Figure 4), including amino acids, peptides and their analogues (23 metabolites), B vitamins (5 metabolites), fatty acid esters (2 metabolites), carbohydrates and their conjugates (2 metabolites), and lipids (5). In contrast, the majority of the downregulated metabolites from the PC-3 cell-derived EVs were non-polar. Asparagine was the only exception (FC(log2) = 0.35038, *p* ≤ 0.021). A list of the 66 metabolites is shown in Table S3.

**Figure 5:**
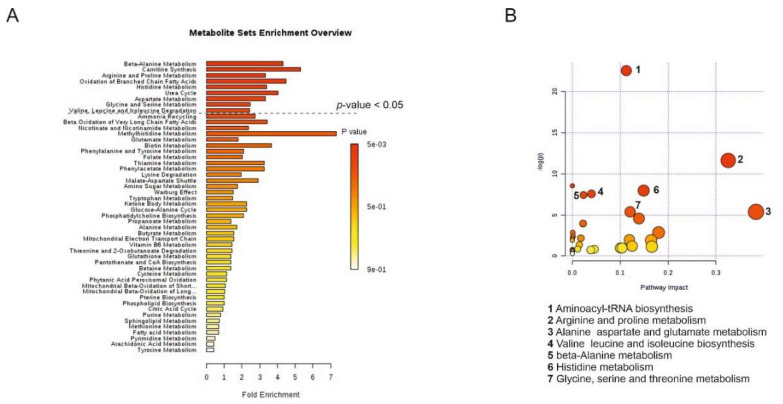
Metabolite pathways that are differentially presented by the cancerous vs. non-cancerous prostate cell line-derived extracellular vesicles (EV). (**A**) A summary plot of the MSEA was implemented using a hypergeometric test (over-representation analysis, ORA, MetaboAnalyst 4.0) of significantly overexpressed metabolites in PC-3 EVs compared to PNT2 EVs. Molecular pathways were ranked according to the probability (1-tailed *p*-value) of finding significantly changed metabolites in a pathway. (**B**) Metabolomics pathway analysis (MetPA) of significantly overexpressed metabolites in PC-3 EVs compared to PNT2 EVs was implemented by the hypergeometric test (over-representation analysis, ORA, MetaboAnalyst 4.0). Matched pathways are arranged by *p*-value (1-tailed Fisher’s exact test) on the *Y*-axis, and the pathway impact values are displayed on the *X*-axis. The color of the node is based on the *p-*value and the radius of the node is based on the pathway impact values. The most affected pathways are indicated by a number and listed in the figure.

Next, the metabolites that were the most upregulated in the EVs derived from PC-3 cells were subjected to a metabolite set enrichment analysis (MSEA) and metabolomics pathway analysis (MetPA). MSEA was used to identify significantly altered pathways based on the upregulated metabolites calculated by over-representation analysis. According to MSEA, several metabolic pathways were significantly altered (*p*-value < 0.05) in the EVs derived from cancerous vs. non-cancerous cells. These included (1) beta-alanine metabolism, (2) carnitine synthesis, (3) arginine and proline metabolism, (4) oxidation of branched-chain fatty acids, (5) histidine metabolism, (6) urea cycle, (7) arginine metabolism, (8) glycine and serine metabolism, and (9) valine, leucine, and isoleucine degradation (Figure 5A). Next, the centrality of an upregulated metabolite was measured in the metabolic network by MetPA filtering the dataset using a false discovery rate (FDR)-adjusted *p*-value < 0.05. MetPA determined that the different metabolites between PC-3- and PNT2-derived EVs belonged to the following topological pathways: (1) aminoacyl-tRNA biosynthesis, (2) arginine and proline metabolism, (3) alanine, aspartate, and glutamate metabolism, (4) valine, leucine, and isoleucine biosynthesis, (5) beta-alanine metabolism, (6) histidine metabolism, and (7) glycine, serine, and threonine metabolism (Figure 5B).

### 2.3. Targeted LC-MS Metabolite Profile Analysis of Cancer EVs

Since the shotgun approach of the non-targeted metabolomics of EVs derived from the PC-3 and PNT2 cell lines revealed a clear difference in the EV metabolome between cancerous and non-cancerous cells, we wanted to determine if any of the altered metabolites would be similarly changed in EVs irrespective of the cancer type. We chose two distinguishable cancers, colon carcinoma and cutaneous T-cell lymphoma (CTCL), represented by cell lines, to determine if the change in the metabolome of their EVs compared to the EVs from their non-cancerous counterpart cells could reveal any shared markers with the EVs derived from the prostate cancer cell line. As the majority of the upregulated EV-derived metabolites in the primary study were polar, only polar metabolites were analyzed by targeted metabolomics using UPLC-MS-MS. The EV metabolome of cancer cell lines (CTCL, Mac-2A and colon carcinoma, RKO) and the corresponding controls (peripheral blood mononuclear cells, PBMC and normal colon epithelia, CCD841) were analyzed in a pairwise manner. A total of 102 polar metabolites (Table S4) were quantitatively measured. Out of the 102 metabolites, 46 were above the lower limit of quantification (in at least one sample group) and were selected for statistical analysis (Table S5). Of the 46 metabolites, 16 were considered to be metabolites of interest based on their significance versus fold-change values, of which 12 were upregulated and four were downregulated in EVs derived from the CTCL cell line compared to non-malignant cultured PBMCs. In the EVs derived from malignant RKO versus the normal colon epithelial cell line CCD841, 10 metabolites were found to be different; four were upregulated and six were downregulated in the corresponding EVs (Table S6).

**Figure 6:**
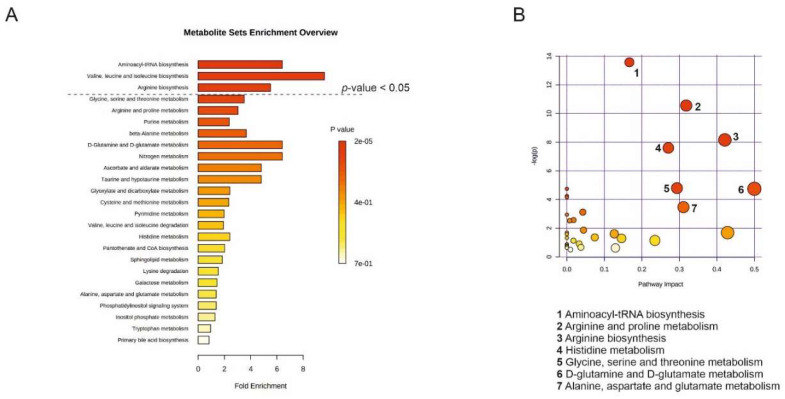
Common metabolite pathways from three different cancer types identified by the alterations between extracellular vesicles (EV) derived from cancerous and non-cancerous cells. (**A**) Summary plot of the MSEA analysis was implemented using a hypergeometric test (over-representation analysis, ORA, MetaboAnalyst 4.0) of significantly overexpressed metabolites in PC-3, CTCL, and RKO EVs compared to their respective control cell-derived EVs. Molecular pathways were ranked according to the probability (1-tailed *p*-value) of finding significantly changed metabolites in the compound list of a certain pathway. (**B**) MetPA analysis of significantly overexpressed metabolites in PC-3, CTCL, and RKO EVs compared to their respective control cell line-derived EVs was implemented using a hypergeometric test (over-representation analysis, ORA, MetaboAnalyst 4.0). Matched pathways are arranged by *p*-value (1-tailed Fisher’s exact test) on the *Y*-axis and pathway impact values on the *X*-axis. The color of the node is based on *p*-value and the radius of the node based on pathway impact values. The most altered metabolic pathways are indicated by numbers and listed in the figure.

Next, we again performed the MSEA and MetPA analyses on the combined list of upregulated metabolites from all three cancer cell lines that were compared pairwise to their non-cancerous counterparts by (Figure 5). MSEA revealed the following three affected pathways common to the three cancers (*p*-value < 0.05): (1) aminoacyl-tRNA biosynthesis, (2) valine, leucine, and isoleucine biosynthesis, and (3) arginine biosynthesis (Figure 6A). MetPA analysis by filtering the dataset using an FDR-adjusted *p*-value < 0.05 revealed that metabolite pathways that differed between the EVs derived from the cancerous and the non-cancerous cell lines were: (1) aminoacyl-tRNA biosynthesis, (2) arginine and proline metabolism, (3) arginine biosynthesis, (4) histidine metabolism, (5) glycine, serine, and threonine metabolism, (6) D-glutamine and D-glutamate metabolism, and (7) alanine, aspartate, and glutamate metabolism (Figure 6B).

**Figure 7:**
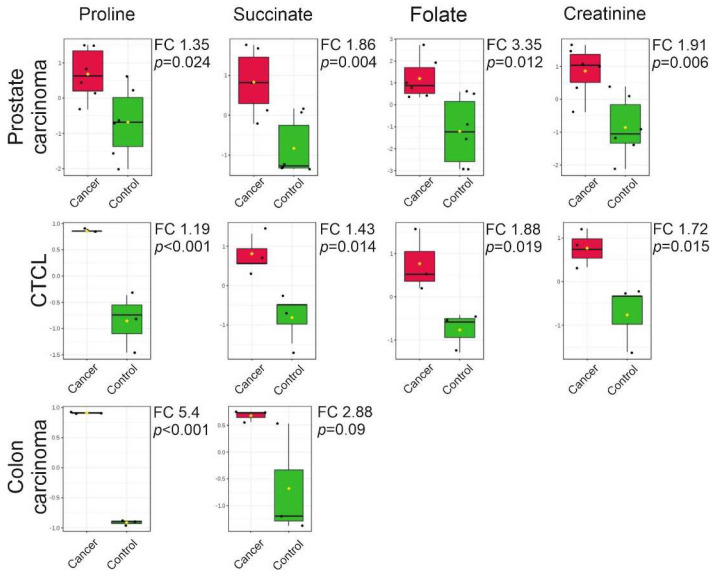
Box plots of the top four metabolites with significant differences in concentration between the extracellular vesicles derived from cancerous cell lines and their respective control cells. Box denotes 25th and 75th percentiles; the line within the box denotes 50th percentile; the whisker denotes standard deviation. FC = log2(fold change). *n* = 6 (prostate), *n* = 3 (CTCL and colon) CTCL = Cutaneous T-cell lymphoma.

The comparison of the datasets revealed that all cancer cell line -derived (prostate, CTCL, colon) EVs share common metabolites that were upregulated when compared with EVs derived from their relevant non-cancerous counterparts (Figure 7). Specifically, all cancer cell line-derived EVs contained elevated levels of proline and succinate based on statistical analysis (significance versus fold-change values). In addition, EVs derived from prostate cancer and CTCL also contained elevated levels of folate and creatinine.

## 3. Discussion

Metabolic reprogramming in cancer cells is one of the hallmarks of cancer, and drugs targeting metabolism have long been applied in cancer treatment [31,32]. Across different cancer types, many metabolic pathways are dysregulated, e.g., energy metabolism, amino acid metabolism, and lipid biogenesis [33,34,35]. The altered metabolism of cancer cells is also reflected in the EV metabolome. Here, we have shown that metabolomics of EVs derived from cancer cells can reveal metabolite signatures indicating cancer and that these metabolites may be shared with cancers originating from different cell types. Importantly, the four cancer-associated metabolites found in this study (proline, succinate, folate, and creatinine) have already been associated with cancers [36,37,38,39]. Overall, EVs derived from cancer cell lines contained greater amounts of amino acids and their derivatives when compared with their non-cancerous counterparts, of which proline had the most prominent change. A known oncometabolite, succinate, was also upregulated in all EVs derived from cancer cell lines compared to control EVs. In the EVs derived from PC-3 and CTCL cells, a greater amount of folate and creatinine was detected compared with their respective non-cancerous controls. This finding suggests that proline, succinate, folate, and creatinine could constitute an EV-based metabolomic fingerprint of cancer.

Previously, metabolomics of plasma and serum samples of cancer patients has been used to identify new cancer biomarkers [36,40,41,42,43]. In several of these studies, an upregulation of amino acids or their derivates has been observed, but no clear consensus on the shared set of amino acids across different cancers has been reached. It may be possible that the isolation of EVs could provide a more homogenous source for metabolites than plasma or serum. In our study, the amino acid proline was 1.35 to 5.4-fold upregulated (log2) in all EVs derived from the cancer cell lines when compared to the controls. Proline is a unique amino acid that has a critical role not only in protein biosynthesis, but also as a regulatory amino acid in cancer metabolism. Proline biosynthesis is altered in cancers, which can support biomass production and proliferation [23,44]. In the proline biosynthesis pathway, Δ-pyrroline-5-carboxylate (P5C) is recycled to proline [45]. In the catabolic reaction, the p53 gene-induced proline dehydrogenase/proline oxidase (PRODH/POX) catalyzes proline back into P5C [46]. In recent studies, it has been shown that the catabolic PRODH/POX pathway produces ATP for autophagy and reactive oxygen species (ROS) for apoptosis, which are both important for cancer progression [47]. Human metastatic breast cancer tissue has also been shown to contain higher levels of PRODH/POX and proline catabolism when compared to the primary breast tumor tissue, suggesting that proline metabolism has a role in metastasis [48]. The human breast cancer-associated gene pyrroline-5-carboxylate reductase (PYCR1) catalyzed the conversion of P5C to proline and its expression was significantly correlated with the invasion and aggressiveness of breast cancer [49]. Although proline levels of plasma samples have been used to identify breast cancer subtypes, the results have been inconclusive. While one study showed that proline levels were upregulated in the more aggressive human epidermal growth factor receptor 2 (HER2)-positive group compared to the HER2-negative group [50], another study revealed that the proline level was decreased in stage-III patients with breast cancer when compared to healthy controls [36]. However, in patients with cervical cancer, plasma levels of proline were shown to be significantly elevated [51].

The second metabolite of interest, succinate, was upregulated 1.86 to 2.88-fold (log2) in EVs derived from all cancer cell lines when compared with their respective controls. Succinate, together with three other oncometabolites (D-2-hydroxyglutarate, L-2-hydroxyglutarate, and fumarate), is a small molecule that accumulates in cancer cells formed by gain-of-function or loss-of-function mutations in genes encoding enzymes of energy metabolism pathways [52]. All four oncometabolites are formed in the mitochondria (tricarboxylic acid cycle) and can cause similar changes in cancer cells, e.g., hypermethylation and pseudohypoxia [6]. Succinate drives tumorigenesis through multiple mechanisms, such as by inducing epigenetic alterations and promoting angiogenesis, invasion, and migration of cancer cells [53,54]. Elevated levels of succinate have been reported in cancer tissues, biofluids of patients with various cancers (e.g., prostate and colorectal cancer [55]), and in hepatocarcinoma, where also proline was found to be upregulated [56] and thus matches our findings from EVs.

Folate (B9 vitamin) was the third cancer-associated metabolite identified in this study and was 1.9-fold upregulated (log2) in CTCL-derived EVs and 3.4-fold upregulated (log2) in prostate cancer cell line-derived EVs. Our results from the non-targeted metabolomics of prostate carcinoma-derived EVs also showed that other B vitamins were upregulated when compared with the EVs derived from the non-cancerous control cell lines. Together with folate, upregulation of pantothenic acid (B5), niacin (B3), thiamine (B1), and pyridoxine (B6) could directly or indirectly enhance one-carbon metabolism by playing a role as co-enzymes and thereby promote cancer progression [57]. One-carbon units are needed for nucleotide synthesis, methylation, and reductive metabolism, which support the high proliferative rate of cancer cells [57]. Most of the earlier B vitamin studies in cancer have focused on assessing whether dietary intake of B vitamins increases cancer risk. Although the results have been inconclusive, a link with B12 and B9 was identified in lung cancer in men [58], breast cancer in women [59], and together with B9, B6, and B2 in colon cancer [60]. Moreover, the folate receptor (FR) was upregulated in ovarian cancer, lung cancer, and breast cancer enabling the enhanced uptake of folate [61]. Recently, the folate receptor FRα has garnered interest as an anti-cancer drug and trials involving FRα-targeted agents and immunotherapies targeted against folate are currently ongoing [61,62].

The fourth metabolite of interest identified in this study, creatinine, was upregulated 1.9-fold (log2) in prostate carcinoma cell line-derived EVs and 1.7-fold upregulated (log2) in CTCL cell line-derived EVs. The creatine kinase/phosphorylcreatine/creatine (CK/PCr/Cr) pathway is known to be involved in the metabolism of the cells with a high-energy demand, such as in proliferating cancer cells [63]. Thus, the role of the CK/PCr/Cr pathway in cancer is well established, but little is known of the role of conversion of creatine to creatinine in cancers. Previously, in prostate cancer, elevated serum creatinine levels have been associated with a more advanced disease stage and decreased survival [64,65]. Increased serum creatinine levels have also been previously associated with vulvar cancer [66], liposarcoma [67], and epithelial ovarian cancer [68].

Using in vitro models for proof-of-concept studies has its limitations. One limitation concerns the control cells chosen for this study. PNT2 cells have been widely characterized for their colony-forming ability, doubling time, and the expression of cytokeratin 19. [69]. Similar to many non-cancerous cells that have been immortalized, PNT2 cells are not completely normal, but they do retain many features of the well-differentiated prostate epithelial cells. Additionally, PNT2 cells are non-tumorigenic when injected in nude mice for a period of 12 months [70]. The control cell lines for colon cancer (CCD841) represent normal colon epithelial cells, yet lacking definitive proof of epithelial origin. The European and US cell collections consider both PNT2 and CCD841 cell lines as normal benign cells. Peripheral blood mononuclear cells (PBMC) were chosen as a control for CTCL primarily to avoid additional separation-induced metabolic (stress) changes in enriched T-cells. In general, ca. 70–90% of PBMCs are lymphocytes and ca. 60–70% of them are T cells. Thus, the comparative cell population represents mostly normal T-cells but not exclusively. Despite the limitations, these control cells present a benign cell type with stable metabolism distinguishing malign cell types apart.

Our findings of the shared EV metabolome reflecting the common metabolic alterations in different cancer types suggest that a pan-cancer EV metabolome exists. Earlier metabolomics studies of cancer patients’ plasma or serum share some of the findings of our study, but have not established consensus over a shared metabolome. By isolating EVs for metabolomics, we could sharpen the focus of the search by (i) avoiding nutrition effects of plasma/serum metabolite profiles and (ii) by enriching cancer cell-derived metabolites through their EVs. For cancers where a tissue biopsy is not an option, analyzing the EV metabolome could offer a new non-invasive tool complementing other diagnostic methods. Thus, we predict that a metabolomic fingerprint of EVs could be developed in the future for cancer detection and treatment monitoring.

## 4. Materials and Methods

### 4.1. Cell Culture

PC-3 (ATCC^®^ CRL-1435, Gaithersburg, MD, USA) prostate carcinoma cell line was grown in Dulbecco’s modified Eagle medium Nutrient Mixture F-12 (DMEM/F12, Gibco, Thermo Fisher Scientific, Waltham, MA, USA) supplemented with Glutamax, penicillin-streptomycin, and 10% of EV-depleted fetal bovine serum (FBS). PNT2 (ECACC^®^ 95012613, Salisbury, UK) represent human prostate epithelial cells, and was grown in RPMI1640 (Gibco, Thermo Fisher Scientific) medium supplemented with Glutamax and penicillin-streptomycin. Cutaneous T-cell lymphoma (CTCL) cell line Mac-2A was a kind gift from Dr. Robert Gniadecki. The Mac-2A cell line was established from the skin tumor of a patient with primary anaplastic large cell lymphoma (pcALCL) [71] and was grown in RPMI1640 (Gibco, Thermo Fisher Scientific) medium supplemented with Glutamax and penicillin-streptomycin. PBMCs were isolated from the buffy coat of healthy blood donors (Finnish Red Cross Blood Service, Helsinki, Finland) with the Lympholyte^®^-H gradient separation method (CL5015, Cedarlane Corporation, Burlington, ON, Canada) according to the manufacturer’s instructions. PBMCs were grown in RPMI1640 medium supplemented with Glutamax, penicillin-streptomycin, 10% EV-depleted FBS, 10 mM HEPES buffer (Lonza, Basel, Switzerland), and 0.5 U/mL IL-2 (11340025, ImmunoTools, Friesoythe, Germany). RKO (ATCC^®^ CRL2577™, Gaithersburg, MD, USA) colon carcinoma cell line and CCD 841 CoN (ATCC^®^ CRL-1790™, Gaithersburg, MD, USA) normal colon epithelial cell line were grown in Minimum Essential Medium (LONBE06-174G, Lonza, Basel, Switzerland) supplemented with Glutamax, penicillin–streptomycin, and 10% EV-depleted FBS. FBS was depleted of serum-derived EVs using the polyethylene glycol (PEG) method. First, a sterile-filtered (0.2-µm filter) 50% (*w*/*v*) PEG stock solution was prepared in 1× Dulbecco’s phosphate-buffered saline (DPBS, Sigma-Aldrich, Damstadt, Germany), protected from light, and stored at 4 °C. The FBS (Gibco, Thermo Fisher Scientific) and PEG (P6667, Sigma-Aldrich, St. Louis, MO, USA) stock solutions were mixed in a 5:1 ratio by inverting 5 to 10 times and incubated for 2 h at 4 °C. The solution was centrifuged for 30 min at 4 °C at 1500× *g* in a swinging-bucket rotor. The supernatant was collected, leaving a layer of 0.5 cm on top of the pellet. The supernatant was sterile filtered (0.1-µm filter) and aliquots were stored at −20 °C. The cell lines were routinely tested for mycobacterial contamination using MycoAlertTM Mycoplasma detection Kit (LT07, Lonza, Basel, Switzerland) and were negative.

### 4.2. EV Isolation Using Differential Ultracentrifugation

EVs were isolated using differential ultracentrifugation as previously described [26] with minor modifications. For each cell line, four isolation rounds were performed as biological replicates. Cell lines were grown for 48 h in a supplemented medium (described above) to obtain 80% confluence for the adherent cells, and optimal cell viability for the suspension cells. For non-targeted metabolomics, the conditioned medium (180 mL) was first centrifuged to remove cell debris and apoptotic bodies at 2500× *g* for 30 min. The supernatant was then centrifuged at 20,000 g^avg^ for 60 min for the 20 K EV pellet and the final supernatant was ultracentrifuged at 110,000 g^avg^ for 2 h at 4 °C to obtain the 110K EV pellet using an Optima LE-80K ultracentrifuge with rotor Ti 50.2, k-factor 143.3 (Beckman Coulter, Brea, CA, USA). For EVs for characterization and targeted metabolomics, the conditioned medium (180 mL) was first centrifuged to remove cell debris and apoptotic bodies at 2500× *g* for 30 min. The supernatant was then centrifuged at 110,000 g^avg^ for 2 h at 4 °C to obtain the EV pellet. The collected EVs were washed with 500 µL of PBS and re-pelleted by ultracentrifugation at 110,000 g^avg^ for 2 h at 4 °C using an Optima MAX-XP (Beckman Coulter, Brea, CA, USA) ultracentrifuge with rotor TLA-55, k-factor 81.3 (Beckman Coulter, Brea, CA, USA). The EV pellets and media controls were then resuspended in 120 μL of DPBS (Gibco, Thermo Fisher Scientific, Waltham, MA, USA) and stored in aliquots at −80 °C until further analysis.

### 4.3. Nanoparticle Tracking Analysis

Particle concentrations of the EV samples (*n* = 4; biological replicates for each cell line) were analyzed by NTA using Nanosight model LM14 (Malvern Panalytical Ltd., Malvern, UK) equipped with blue (404 nm, 70 mW) laser and SCMOS camera. The samples were diluted in 0.1-µm filtered (Millex VV, Merck Millipore, Dorset, UK) DPBS to obtain 40 to 100 particles/view, and five 30-s videos were recorded using camera level 14 with an automatic temperature setting of 22 °C. The data were analyzed using NTA software 3.0 with detection threshold 5 and screen gain at 10 to track as many particles as possible with minimal background.

### 4.4. Western Blotting

WB analyses of cell line-derived EVs were performed using 1.0E10 EV particles per lane. Samples were boiled at 95 °C for 5 min in Laemmli Sample Buffer (BioRad, Sweden) with 2-mercaptoethanol and with additional sodium dodecyl sulfate (SDS, total 8%). In total, 12% SDS-PAGE gels were run and proteins were transferred onto nitrocellulose membranes using a wet transfer system. Membranes were incubated overnight at 4 °C with monoclonal antibodies against TSG101 (1:100, MA1-23296, ThermoFisher Scientific, Waltham, MA, MA, USA), RAB5 (1:100, PA5-29022, ThermoFisher Scientific, Waltham, MA, USA), TOM20 (1:1000, 42406, Cell Signaling, Leiden, The Netherlands), followed by incubation with anti-rabbit (PO448, Dako, Denmark) or anti-mouse (PO447, Dako, Denmark) HRP-conjugated secondary antibody for 1 h at room temperature.

### 4.5. Immunoelectron Microscopy

Anti-CD63 staining for IEM from EV samples was prepared as described by Puhka et al. [16], including the paraformaldehyde (PFA) fixation with the additional immunostaining step. Briefly, after loading to 200 mesh copper grids, the samples were blocked with 0.5% BSA in 0.1 M Na_2_PO_4_ buffer (pH 7.0) followed by a 45-min incubation at room temperature with anti-CD63 (1:100, 11-343-C100, Exbio, Praha, Czech Republic) antibody and 10-nm gold-conjugated goat-anti-mouse-IgG secondary antibody (1/80 dilution, BBI Solutions, Cardiff, UK). Samples were washed with the NaPO_4_ buffer and deionized water. Samples were viewed with transmission electron microscopy Jeol JEM-1400 (Jeol Ltd., Tokyo, Japan). Images were taken with a Gatan Orius SC 1000B CCD camera (Gatan Inc., Pleasanton, CA, USA).

### 4.6. Non-Targeted Liquid Chromatography-Mass Spectrometry Metabolite Analysis

EVs isolated from three isolation rounds of the prostate cancer cell line PC-3 (*n* = 6) and the benign prostate epithelial cell line PNT2 (*n* = 6) were analyzed for their metabolome with LC-qTOF-MS as described previously [28]. In short, metabolites were extracted from the EV samples in triplicates (biological replicates) with acetonitrile. The samples were then centrifuged 16,000 × *g* for 10 min and the supernatants were filtered with 0.2-μm Acrodisc^®^ Syringe filters with PFTE membrane (Pall Corporation, Ann Arbor, MI, USA). For quality control, small aliquots from every individual sample were pooled together. The extracted metabolites were then further analyzed using LC-MS instrumentation consisting of a 1290 LC system, a Jetstream electrospray ionization (ESI) source, and a 6540 UHD accurate-mass qTOF spectrometer (Agilent Technologies, Wallbronn, Karlsruhe, Germany). The samples were analyzed using both reverse phase (RP, Zorbax Eclipse XDB-C 18 column, Agilent Technologies, Wallbronn, Karlsruhe, Germany) and hydrophilic interaction chromatography (HILIC, Acquity UPLC BEH Column, Waters Corporation, Milford, MA, USA) to maximize coverage of metabolites. For data acquisition, the mass range was set to 20-1600 amu with an acquisition rate of 1.67 spectra/s. For automatic MS/MS spectrums from every precursor scan cycle, four ions with the highest intensities were selected for fragmentation. Collision energies used for fragmentation were 10, 20, and 40 eV. Data acquisition was conducted with MassHunter Acquisition B.04.00 (Agilent Technologies, Wallbronn, Karlsruhe, Germany). The raw MS/MS data were collected using the vendor’s software MassHunter Qualitative Analysis B.05.00 (Agilent Technologies, Wallbronn, Karlsruhe, Germany). To remove insignificant metabolite features, following inclusion criteria were used: the molecular feature needs to be detected in minimum of 2/3 of the samples in at least one replicate group. The raw MS/MS data were deconvoluted, aligned, and identified with MS DIAL. Identification of metabolites was verified by screening the mass and MS/MS fragmentation spectra in the Human Metabolome Database HMDB and LIPID MAPS. The fragmentation pattern of the metabolites was compared with that of the standards of the same molecules when available or found in databases and verified by earlier literature.

### 4.7. Targeted Liquid Chromatography-Mass Spectrometry Metabolite Analysis

EVs derived from three isolation rounds of RKO, CCD840, Mac-2A, and PBMC cells (*n* = 3) were applied to targeted analysis of 102 metabolites (Table S3) using ultra-performance liquid chromatography-tandem mass spectrometer (UPLC-MS-MS, ACQUITY UPLC^®^ with Xevo TQ-S—tandem quadrupole mass spectrometer, Waters Corporation, Milford, MA, USA) as previously described [16,70]. In short, a labeled internal standard mixture was added to the samples prior to the metabolite extraction. Next, samples were collected into a 96-well plate, sealed, and centrifuged at 4000 rpm for 15 min at 4 °C and placed in an auto-sampler of the liquid chromatography system for sample injection. Metabolite analysis was performed on an ACQUITY UPLC-MS-MS system (Waters Corporation, Milford, MA, USA). All metabolites were separated using a 2.1 × 100 mm Acquity 1.7 µ BEH amide HILIC column (Waters Corporation, Milford, MA, USA) maintained at 45 °C. The total run time was 14.5 min, including 2.5 min equilibration at a flow rate of 600 µL/min. The detection system, a Xevo^®^ TQ-S tandem triple quadrupole mass spectrometer (Waters Corporation, Milford, MA, USA), was operated in both positive and negative polarities with polarity switching time of 20 msec. Multiple Reaction Monitoring (MRM) acquisition modes were selected for quantification of the metabolites with an individual span time of 0.1 s given in their MRM channels. The dwell time was automatically calculated by the software by the region of the retention time window, a number of MRM functions, and also depending on the number of data points required to form the peak. MassLynx 4.1 software was used for data acquisition, handling, and instrument control. Data processing was performed with TargetLynx software and metabolites were quantified using area ratio (area of metabolite/area of IS) standards and external calibration curves. A peak response giving a signal to noise (S/N) ratio of <3 was taken to below the limit of detection. For the calculation of concentration averages, concentrations below this limit were assigned as zeros. The lower limit of quantification was defined as the lowest concentration on the calibration curve corresponding to S/N of >6.

### 4.8. Statistical Analysis

For all analyses, the data from a cancer cell line were compared with the data from the respective control cell line or cells. To compare NTA data, the average and standard deviation were calculated for each sample type (*n* = 4). Metabolomics data analysis was performed using Metaboanalyst v4.0 [71]. Data from non-targeted metabolite analysis of PC-3 and PNT2-derived 20K and 110K EVs were combined, normalized using quantile method, log2 transformed, and scaled using Pareto scaling prior statistical analysis. Before statistical analysis from targeted metabolite analysis, results were manually verified to ensure the shape and integration of the peak of each metabolite in all samples. The concentration of each metabolite was verified to be above the value of “below lower limit of quantification” (BLLOQ), normalized to the particle concentration from NTA (fmol/particle) and prior to the statistical analysis further normalized using quantile method, log2 transformation, and auto-scaling.

### 4.9. Metabolic Pathway Analysis

Pathway analysis was performed on the metabolites that were found to be significantly upregulated in the cancer EVs compared to the respective control EVs (*p*-value < 0.05 and fold change > 2). Pathway information for each metabolite was extracted from either HMDB [72] or Kyoto Encyclopedia of Genes and Genomes (KEGG) [73]. A more detailed analysis of the identified metabolites and pathways and networks of interest was performed by MetaboAnalyst v4.0 [74]. To exclude possible carry-over effects of media, a comparison of media components was conducted for PC-3 and PNT2.

## 5. Conclusions

In the present study, we showed that the altered metabolism in cancer cells affects the EV metabolome, and by metabolomics, it is possible to distinguish cancer cell -derived EVs from control cell -derived EVs in in vitro settings. Comparing the EV metabolome of PC-3 cell line -derived EVs to PNT2 cell line -derived EVs in an untargeted manner resulted in 41 upregulated and 25 downregulated metabolites. The majority of cancer-associated EV metabolites were soluble, e.g., amino acids and their derivates, and B vitamins. This finding was further corroborated by analyzing two additional cancer cell line -derived EVs (CTCL and colon carcinoma) and their control EVs in a targeted manner. Taken together, the findings suggest that EVs reflect the metabolic status of the cancer cells and different cancer types share a similar EV metabolome that could be used for diagnostics in the future. The marker panel established in this study (proline, succinate, folate, and creatinine) was shared by different cancer types and warrants further investigation.

## Supplementary Materials

The following are available online at https://www.mdpi.com/2072-6694/12/11/3292/s1, Table S1. Non-targeted metabolomics of PC-3 and PNT2 cell line-derived EVs; Table S2. Composition of media for PC-3 and PNT2 cell lines; Table S3. Metabolites differing significantly between PC-3 and PNT2 cells lines measured from EVs; Table S4. Metabolites measured from Mac2A, PBMC, RKO, and CCD841 cell line-derived EVs in a targeted manner; Table S5. Metabolites measured in a targeted manner and selected for statistical analysis; Table S6. Metabolites showing a significant difference between Mac2A and PBMC-derived EVs and RKO and CCD841-derived EVs, respectively. Figure S1. Western blot original figures of PC-3, PNT2, Mac2A, PBMC, RKO, and CCD841 cell line-derived EVs probed with TSG101, RAB5, and TOM20 antibodies. Molecular weight is marked by black arrow.
