# Supplementary material for: Cancer Alters the Metabolic Fingerprint of Extracellular Vesicles
Source: Cancers (Basel). 2020 Nov 6;12(11):3292. doi: 10.3390/cancers12113292 (PMC7694806; doi:10.3390/cancers12113292)
Supplement: Supplementary file 1 [file cancers-12-03292-s001.zip › Table S2.pdf]

DMEM/F12 media for PC-3 cells	
Components	mM
Amino Acids	
Glycine	0.25
L-Alanine	0.049999997
L-Alanyl-L-Glutamine	2.497696
L-Arginine hydrochloride	0.69905216
L-Asparagine-H2O	0.05
L-Aspartic acid	0.05
L-Cysteine hydrochloride-H2O	0.09977272
L-Cystine 2HCl	0.09996805
L-Glutamic Acid	0.05
L-Histidine hydrochloride-H2O	0.14990476
L-Isoleucine	0.41580153
L-Leucine	0.45076334
L-Lysine hydrochloride	0.4986339
L-Methionine	0.11570469
L-Phenylalanine	0.2150303
L-Proline	0.15
L-Serine	0.25
L-Threonine	0.44915968
L-Tryptophan	0.04421569
L-Tyrosine disodium salt dihydrate	0.21375479
L-Valine	0.4517094
Vitamins	
Biotin	1.4344263E-5
Choline chloride	0.06414285
D-Calcium pantothenate	0.0046960167
Folic Acid	0.0060090707
Niacinamide	0.016557377
Pyridoxine hydrochloride	0.009859223
Riboflavin	5.824468E-4
Thiamine hydrochloride	0.0064391694
Vitamin B12	5.0184503E-4
i-Inositol	0.07
Inorganic Salts	
Calcium Chloride (CaCl2) (anhyd.)	1.0504504
Cupric sulfate (CuSO4-5H2O)	5.2E-6
Ferric Nitrate (Fe(NO3)3"9H2O)	1.2376238E-4
Ferric sulfate (FeSO4-7H2O)	0.0015
Magnesium Chloride (anhydrous)	0.30147368
Magnesium Sulfate (MgSO4) (anhyd.)	0.407
Potassium Chloride (KCl)	4.1573334
Sodium Bicarbonate (NaHCO3)	29.02381
Sodium Chloride (NaCl)	120.68104
Sodium Phosphate dibasic (Na2HPO4) anhydrous	0.50014085
Sodium Phosphate monobasic (NaH2PO4-H2O)	0.45289856
Zinc sulfate (ZnSO4-7H2O)	0.0015
Other Components	
D-Glucose (Dextrose)	17.505556
Hypoxanthine Na	0.015031448
Linoleic Acid	1.4999999E-4
Lipoic Acid	5.097087E-4
Phenol Red	0.021519661
Putrescine 2HCl	5.031056E-4
Sodium Pyruvate	0.5
Thymidine	0.0015082645

RPMI1640 media for PNT2 cells	
Components	mM
Amino Acids	
Glycine	0.13333334
L-Alanyl-Glutamine	2.0552995
L-Arginine	1.1494253
L-Asparagine	0.37878788
L-Aspartic acid	0.15037593
L-Cystine	0.20833333
L-Glutamic Acid	0.13605443
L-Histidine	0.09677419
L-Hydroxyproline	0.15267175
L-Isoleucine	0.3816794
L-Leucine	0.3816794
L-Lysine hydrochloride	0.21857923
L-Methionine	0.10067114
L-Phenylalanine	0.09090909
L-Proline	0.17391305
L-Serine	0.2857143
L-Threonine	0.16806723
L-Tryptophan	0.024509804
L-Tyrosine	0.110497236
L-Valine	0.17094018
Vitamins	
Biotin	8.1967213E-4
Choline chloride	0.021428572
D-Calcium pantothenate	5.24109E-4
Folic Acid	0.0022675737
Niacinamide	0.008196721
Para-Aminobenzoic Acid	0.00729927
Pyridoxine hydrochloride	0.004854369
Riboflavin	5.319149E-4
Thiamine hydrochloride	0.002967359
Vitamin B12	3.690037E-6
i-Inositol	0.19444445
Inorganic Salts	
Calcium nitrate (Ca(NO3)2 4H2O)	0.42372882
Magnesium Sulfate (MgSO4-7H2O)	0.40650406
Potassium Chloride (KCl)	5.3333335
Sodium Bicarbonate (NaHCO3)	23.809525
Sodium Chloride (NaCl)	103.44827
Sodium Phosphate dibasic (Na2HPO4) anhydrous	5.633803
Other Components	
D-Glucose (Dextrose)	11.111111
Glutathione (reduced)	0.0032573289
Phenol Red	0.013283741
