# Supplementary material for: Cancer Alters the Metabolic Fingerprint of Extracellular Vesicles
Source: Cancers (Basel). 2020 Nov 6;12(11):3292. doi: 10.3390/cancers12113292 (PMC7694806; doi:10.3390/cancers12113292)
Supplement: Supplementary file 1 [file cancers-12-03292-s001.zip › Table S3.pdf]

Supplementary table 3.

Table of the differential metabolites by their significance versus fold-change –values in EVs derived from PC-3 cell line compared to PNT2 cell line. The metabolites of the 20K and 110K EVs of both cell lines were combined.

Metabolite	log2(FC)	p-value
Threonine	4,291	0,001334
Pyridoxine	3,4913	0,000335
Folic acid	3,3478	0,011708
Lysine	3,3473	4,54E-05
Glutamine	3,2707	0,000633
Phenylalanine	3,1977	0,000558
Carnosine	2,9964	0,002953
Tryptophan	2,8058	0,00073
Thiamine cation	2,7628	0,000723
Niacinamide	2,7474	0,00284
PC35:0	2,7396	5,65E-06
PE36:2	2,6761	0,004276
Valine	2,6648	0,000423
Mannitol	2,4872	0,014692
Serotonin	2,4291	0,010827
Pantothenic acid	2,3868	0,000646
Acetylcarnitine	2,281	0,000501
Glutamine	2,2004	0,005985
PC17:0/18:1	2,1895	8,37E-05
Trimethyl-L-ysine	2,1409	0,004703
Isobutyryl carnitine	2,1354	0,002631
Glycine betaine	2,039	0,006382
Creatinine	1,9129	0,005874
Succinic acid	1,8641	0,004406
Histidine	1,8509	0,005703
1-methyl-histidine	1,8411	0,035906
Isoleucine	1,828	0,011466
PC33:0	1,7748	0,000821
Propionylcarnitine	1,7667	0,014771
Leucine	1,6823	0,012445
Citrulline	1,6397	0,034158
Glutamate	1,5881	0,03004
Phosphocholine	1,5722	0,064037
Dimethyarginine	1,4828	0,057635
Creatine	1,4812	0,027922
L-arginine	1,4762	0,044143
Carnitine	1,423	0,034786
Penicillin G	1,4061	0,016697
Proline	1,351	0,024089
4-acetamidobutanoate	1,298	0,053739
3-methyl-histidine	1,2547	0,042929

Metabolite	log2(FC)	p-value
LysoPC17:0/00	-1,0915	0,012378
PC18:1/18:0	-1,1032	0,008292
PC36:1	-1,1204	0,014118
SM34:0	-1,129	0,002712
PE18:1/18:1	-1,1574	0,000132
PC36:5	-1,1659	0,032435
LysoPC18:0/00	-1,2028	0,017827
PC37:4	-1,2143	0,05012
LysoPE20:4/00	-1,2464	0,010259
PE18:0/20:4	-1,2751	0,020424
PE18:1/22:6	-1,3053	0,000179
LysoPE22:6/00	-1,4377	0,028173
Asparagine	-1,513	0,021049
PC37:2	-1,5421	0,016206
PC18:0/20:3	-1,5604	0,046818
PC34:0	-1,5973	0,057397
PC34:3	-1,7	0,006729
LysoPC00/18:0	-1,7129	0,004576
Oleamide	-1,8412	0,067512
PC34:4	-2,0692	0,020672
PE16:0/22:5	-2,3261	0,01515
PC38:2	-2,5503	0,002334
PE18:0/22:6	-2,737	0,007763
PE18:0/18:1	-2,7437	0,00063
SM36:1	-3,0849	0,001575
