# Supplementary material for: Cancer Alters the Metabolic Fingerprint of Extracellular Vesicles
Source: Cancers (Basel). 2020 Nov 6;12(11):3292. doi: 10.3390/cancers12113292 (PMC7694806; doi:10.3390/cancers12113292)
Supplement: Supplementary file 1 [file cancers-12-03292-s001.zip › Table S4.pdf]

	metabolite	group	HMDB
**1**	NAD	(5'->5')-dinucleotides	HMDB0000902
**2**	Pantothenic Acid	Alcohols and polyols	HMDB0000210
**3**	1-methylhistamine	Amines	HMDB0000898
**4**	Spermidine	Amines	HMDB0001257
**5**	Carnitine	Amines	HMDB00898
**6**	Myoinositol	Amines	HMDB0000211
**7**	Creatine	Amino acids, peptides, and analogues	HMDB0000064
**8**	Creatinine	Amino acids, peptides, and analogues	HMDB0000562
**9**	Cystathionine	Amino acids, peptides, and analogues	HMDB0000099
**10**	DimethylGlycine	Amino acids, peptides, and analogues	HMDB0000092
**11**	Folic Acid	Amino acids, peptides, and analogues	HMDB0000121
**12**	Gamma-Glutamylcysteine	Amino acids, peptides, and analogues	HMDB0001049
**13**	Glutamine	Amino acids, peptides, and analogues	HMDB0003423
**14**	Glutathione	Amino acids, peptides, and analogues	HMDB0000125
**15**	Glycine	Amino acids, peptides, and analogues	HMDB0000123
**16**	Guanidinoacetic Acid	Amino acids, peptides, and analogues	HMDB0000128
**17**	Histidine	Amino acids, peptides, and analogues	HMDB0000177
**18**	Homocysteine	Amino acids, peptides, and analogues	HMDB0000742
**19**	Homoserine	Amino acids, peptides, and analogues	HMDB0000719
**20**	Hydroxyproline	Amino acids, peptides, and analogues	HMDB0000725
**21**	Isoleucine	Amino acids, peptides, and analogues	HMDB0000172
**22**	Leucine	Amino acids, peptides, and analogues	HMDB0000687
**23**	L-Glutamic Acid	Amino acids, peptides, and analogues	HMDB0000148
**24**	L-Methionine	Amino acids, peptides, and analogues	HMDB0000696
**25**	Lysine	Amino acids, peptides, and analogues	HMDB0003405
**26**	Phenylalanine	Amino acids, peptides, and analogues	HMDB0000159
**27**	Proline	Amino acids, peptides, and analogues	HMDB0003411
**28**	Symmetric dimethylarginine	Amino acids, peptides, and analogues	HMDB0003334
**29**	Tryptophan	Amino acids, peptides, and analogues	HMDB0000929
**30**	Tyrosine	Amino acids, peptides, and analogues	HMDB0000158
**31**	Valine	Amino acids, peptides, and analogues	HMDB0000883
**32**	Threonine	Amino acids, peptides, and analogues	HMDB0000167
**33**	Aminodipic Acid	Amino acids, peptides, and analogues	HMDB0000510
**34**	2-Aminoisobutyric acid	Amino acids, peptides, and analogues	HMDB0001906
**35**	Arginine	Amino acids, peptides, and analogues	HMDB0000517
**36**	Asparagine	Amino acids, peptides, and analogues	HMDB0033780
**37**	L-Aspartic acid	Amino acids, peptides, and analogues	HMDB0000191
**38**	Asymmetric dimethylarginine	Amino acids, peptides, and analogues	HMDB0001539
**39**	Serine	Amino acids, peptides, and analogues	HMDB0000187
**40**	Betaine	Amino acids, peptides, and analogues	HMDB0000043
**41**	GABA	Amino acids, peptides, and analogues	HMDB0000112

**42**	Citrulline	Amino acids, peptides, and analogues	HMDB0000904
**43**	Ornithine	Amino acids, peptides, and analogues	HMDB0000214
**44**	Alanine	Amino acids, peptides, and analogues	HMDB0000056
**45**	Trimethylamine-N-Oxide	Aminoxides	HMDB0000925
**46**	3-Hydroxyanthranilic acid	Benzoic acids and derivatives	HMDB0001476
**47**	Hippuric acid	Benzoic acids and derivatives	HMDB0000714
**48**	Glycocholic Acid	Bile acids, alcohols and derivates	HMDB0000138
**49**	Taurochenodeoxycholic Acid	Bile acids, alcohols and derivatives	HMDB0000951
**50**	Taurocholic Acid	Bile acids, alcohols and derivatives	HMDB0000036
**51**	Chenodeoxycholic Acid	Bile acids, alcohols and derivatives	HMDB0000518
**52**	Cholic Acid	Bile acids, alcohols and derivatives	HMDB0000619
**53**	IMP	Carbohydrates and carbohydrate conjugates	HMDB0011681
**54**	Sorbitol	Carbohydrates and carbohydrate conjugates	HMDB0000247
**55**	Succinate	Carbohydrates and carbohydrate conjugates	HMDB0000254
**56**	Sucrose	Carbohydrates and carbohydrate conjugates	HMDB0000258
**57**	D-Glucuronic acid	Carbohydrates and carbohydrate conjugates	HMDB0000127
**58**	D-Ribose 5-phosphate	Carbohydrates and conjugates	HMDB0001548
**59**	Glyceraldehyde	Carbohydrates and conjugates	HMDB0001051
**60**	L-Kynurenine	Carbonyl compounds	HMDB0000684
**61**	3-OH-DL-KYNURENINE	Carbonyl compounds	HMDB0000732
**62**	cGMP	Cyclic purine nucleotides	HMDB0011629
**63**	cAMP	Cyclic purine nucleotides	HMDB0011616
**64**	Decanoylcarnitine	Fatty acid esters	HMDB0000651
**65**	Hexanoylcarnitine	Fatty acid esters	HMDB0000756
**66**	Octanoylcarnitine	Fatty acid esters	HMDB0000791
**67**	Propionylcarnitine	Fatty acid esters	HMDB0000824
**68**	Acetylcarnitine	Fatty acid esters	HMDB0000201
**69**	Isovalerylcarnitine	Fatty acid esters	HMDB0000688
**70**	Isobutyrylcarnitine	Fatty acids and conjugates	HMDB0062556
**71**	Carnosine	Hybrid peptides	HMDB0000033
**72**	Allantoin	Imidazoles	HMDB0000462
**73**	5-Hydroxyindole-3-acetic acid	Indolyl carboxylic acids and derivatives	HMDB0000763
**74**	Normetanephrine	Methoxyphenols	HMDB0000819
**75**	Taurine	Organosulfonic acids and derivatives	HMDB0000251
**76**	Homogentisic acid	Phenylacetic acids	HMDB0000130
**77**	Phosphoethanolamine	Phosphate esters	HMDB0000224
**78**	Neopterin	Pterins and derivates	HMDB0000845
**79**	Guanosine	Purine nucleosides	HMDB0000133

**80**	Inosine	Purine nucleosides	HMDB0000195
**81**	Adenosine	Purine nucleosides	HMDB0000050
**82**	Xanthosine	Purine nucleosides	HMDB0000299
**83**	Adenine	Purines and purine derivatives	HMDB00034
**84**	Xanthine	Purines and purine derivatives	HMDB0000292
**85**	Hypoxanthine	Purines and purine derivatives	HMDB0000157
**86**	4-Pyridoxic Acid	Pyridinecarboxylic acids and derivatives	HMDB0000017
**87**	Niacinamide	Pyridinecarboxylic acids and derivatives	HMDB0001488
**88**	Nicotinic Acid	Pyridinecarboxylic acids and derivatives	HMDB0001488
**89**	Pyridoxine	Pyridoxines	HMDB0000239
**90**	2-deoxycytidine	Pyrimidine 2'-deoxyribonucleosides	HMDB0000014
**91**	2-deoxyuridine	Pyrimidine deoxyribonucleotides	HMDB0001191
**92**	Cytidine	Pyrimidine nucleosides	HMDB0000089
**93**	UDP-Glucose	Pyrimidine nucleotide sugars	HMDB0000286
**94**	Cytosine	Pyrimidines and pyrimidine derivatives	HMDB0000630
**95**	Orotic acid	Pyrimidines and pyrimidine derivatives	HMDB0000226
**96**	Uracil	Pyrimidines and pyrimidine derivatives	HMDB0000300
**97**	Cotinine	Pyrrolidinylpyridines	HMDB0001046
**98**	Choline	Quaternary ammonium salts	HMDB0000097
**99**	Kynurenic Acid	Quinoline carboxylic acids	HMDB0000715
**100**	AMP	Ribonucleoside 3'-phosphates	HMDB0003540
**101**	Acetoacetic acid	Short-chain keto acids and derivatives	HMDB0000060
**102**	L-5-Hydroxytryptophan	Tryptamines and derivates	HMDB0000472
