# Supplementary material for: Cancer Alters the Metabolic Fingerprint of Extracellular Vesicles
Source: Cancers (Basel). 2020 Nov 6;12(11):3292. doi: 10.3390/cancers12113292 (PMC7694806; doi:10.3390/cancers12113292)
Supplement: Supplementary file 1 [file cancers-12-03292-s001.zip › Table S6.pdf]

Supplementary table 6A. Metabolites that differ significantly (FC>2, $p \leq 0.05$) between CTCL EVs and PBMC EVs.

Metabolite	log2(FC)	p-value	Upregulated in
**Uracil**	3.9	0.012	CTCL
**Gamma-Glutamylcysteine**	2.7	<0.001	CTCL
**Folic Acid**	1.9	0.019	CTCL
**Creatinine**	1.7	0.015	CTCL
**Hydroxyproline**	1.6	<0.001	CTCL
**Myoinositol**	1.5	<0.001	CTCL
**Succinate**	1.4	0.013	CTCL
**Symmetric dimethylarginine**	1.3	0.004	CTCL
**Proline**	1.2	<0.001	CTCL
**Guanosine**	-4.2	0.004	PBMC
**Adenosine**	-5.0	0.018	PBMC
**Taurine**	-8.9	<0.001	PBMC
**Hypoxanthine**	-8.3	<0.001	PBMC

Supplementary table 6B. Metabolites that differ significantly (FC>2, $p \leq 0.05$) between RKO EVs and CDD488 EVs.

Metabolite	log2(FC)	p-value	Upregulated in
**Proline**	5.4	<0.001	RKO
**L-Methionine**	5.0	<0.001	RKO
**Guanidinoacetic Acid**	-4.3	<0.001	CCD488
**3-Hydroxyanthranilic Acis**	-4.5	<0.001	CCD488
**Gamma-Glutamylcysteine**	-4.7	<0.001	CCD488
**L-Kynurenine**	-4.9	0.047	CCD488
